# The Cone Beam Computed Tomography Evaluation of Cortical Bone Plate after Piezocision-Assisted Orthodontic Upper Arch Expansion: A Case Series

**DOI:** 10.3390/ma14226967

**Published:** 2021-11-18

**Authors:** Maria Julia Pietruska, Emilia Waszkiewicz, Anna Skurska, Eugeniusz Sajewicz, Ewa Dolińska, Małgorzata Pietruska

**Affiliations:** 1 Independent Researcher, ul. Waszyngtona 1/34, 15-269 Białystok, Poland; maria.pietruska@gmail.com (M.J.P.); milkanet@interia.pl (E.W.); 2 Department of Periodontal and Oral Mucosa Diseases, Medical University of Białystok, ul. Waszyngtona 13, 15-269 Białystok, Poland; annaskurska@wp.pl (A.S.); edudziuk@poczta.onet.pl (E.D.); 3 Faculty of Mechanical Engineering, Institute of Biomedical Engineering, Białystok University of Technology, ul. Wiejska 45c, 15-351 Białystok, Poland; e.sajewicz@pb.edu.pl

**Keywords:** cone beam computed tomography, oral surgery

## Abstract

Background: The purpose of the study was to evaluate cone beam computed tomography (CBCT) after piezocision-assisted orthodontic maxillary arch expansion. Methods: Forty CBCT images of 20 patients taken before and after treatment were included in the study. The following radiographic parameters were measured: buccal/palatal bone plate thickness measured in three locations, 0.5 mm, 3.5 mm, and 5 mm from the margin of alveolar process; cemento-enamel junction-crest distance (CEJ-C) measured at buccal (CEJ-B) and palatal/lingual (CEJ-P) aspects. Results: After treatment there were insignificant changes in CEJ-C and thickness of buccal/palatal plates for all the dental groups except for incisors and premolars. CEJ-B increased by 1.43 mm on premolars and CEJ-P by 1.65 mm on incisors and by 0.31 mm on premolars. On the incisors, the buccal plate width increased significantly, by 0.2 mm and 0.44 mm at 3.5-mm and 5-mm measurement points. On premolars, the buccal plate width decreased in three measuring points by 0.27 mm, 0.37 mm, and 0.25 mm. Conclusions: Piezocision-assisted orthodontic maxillary arch expansion does not cause evident negative changes of cortical plates except for the premolar region. Therefore, premolars may be at greater risk of buccal plate loss than other teeth.

## 1. Introduction

Evaluation of the soft and hard periodontal tissues’ status is crucial while adequately diagnosing and making a corresponding orthodontic treatment plan. It seems that the thin periodontal phenotype is a meaningful risk factor for gingival recessions’ appearance after dental arch expansion [1,2]. Gingival recession is a consequence of a decrease in cortical bone thickness and the formation of bone dehiscence/fenestration after orthodontic tooth shifting/tipping [1,3,4,5,6].

To minimalize the probability of complications in the course of orthodontic treatment or in the retention phase, additional surgical procedures are proposed such as minimally invasive corticotomy-piezocision. The intention of the procedure is to make cuts between roots in the buccal plate with the use of a piezosurgery device after the tunnel approach [7,8]. It has been shown that bone injury leads to significant acceleration of bone turnover by increasing the number of osteoclasts, causing transient osteopenia and, consequently, allowing tooth movement with an adjacent demineralized bone matrix. This sequence of reactions, named regional acceleratory phenomena (RAP), reduces bone resistance to tooth movement, which in turn results in faster and safer tooth movement [9,10,11,12,13,14]. The potential benefits of corticotomy make this procedure an increasingly common part of orthodontic treatment [14,15,16,17,18].

Contemporary planning of orthodontic treatment, especially assisted with corticotomy, is associated with the necessity of performing a detailed examination, including cone beam computed tomography (CBCT). CBCT allows determining precisely the width of the alveolar process, the thickness of the buccal bone plate, or location and angulation of roots in the early diagnostic stage. It enables the evaluation of risk factors imperceptible in clinical examination or 2D radiographic examination and, according to this extended data, developing an optimized treatment plan. This examination also gives the opportunity to control the safety of the treatment, allowing us to visualize the position of the roots before removing the orthodontic appliances. The CBCT examination seems to be even more reasonable in patients in whom buccal tooth movement has been performed, especially in those with thin phenotype [19,20,21,22]. It also allows analyzing many other aspects of orthodontic treatment, such as assessment of skeletal growth pattern, severity of tooth impaction, and upper airway evaluation for possible obstructions [23].

Despite its undoubted advantages, CBCT examination is not routinely performed for orthodontic indications. Therefore, there is little data in the available literature regarding changes in alveolar width or cortical bony plates’ width after orthodontic treatment, especially after corticotomy-assisted orthodontic treatment. Knowledge of possible changes in radiographic parameters could be used to assess treatment risks resulting from the possibility of bone dehiscence and subsequent gingival recession after arch expansion. Considering the above, the purpose of the study was to evaluate cortical bone plate (buccal/palatal plate width, occurrence and extent of bone dehiscence) in cone beam computed tomography after piezocision-assisted orthodontic maxillary arch expansion.

## 2. Materials and Methods

### 2.1. Study Design and Population

The study was designed as a single-center, case series study. The study was based on 40 CBCT images of 20 patients treated in a Private Practice in Białystok, Poland, between June 2014 and June 2018. The study was compliant with the Helsinki Declaration of 1975, as revised in 2000, and reviewed and approved by the Ethical Committee of Medical University of Białystok (R-I-002/472/2018). Each participant gave informed consent for the study.

The surgical procedure was performed by one operator (A.S.), and the same for the orthodontic treatment (E.W.) and CBCT evaluation (M.J.P.).

### 2.2. Patient Selection

Inclusion criteria was as follows: adult (>18 years old), non-smoker, generally healthy, with malocclusion with transverse maxillary deficiency with indications for upper arch expansion. Exclusion criteria were the following diseases or conditions: periodontal disease; bisphosphonate and long-term corticosteroid or nonsteroidal anti-inflammatory therapy; current therapy with anti-epileptic drugs, contraceptives, estrogen, antihistamine drugs, calcitonin, vitamin D; pregnancy, breast feeding; previous orthodontic treatment and root resorption. Within the study group, the following malocclusions were diagnosed: Class I with crowding, eight patients; Class II Division, 1–3 patients; Class II Division 1 with anterior open bite, one patient; Class II Division, 2–3 patients; Class III skeletal relationship, two patients; anterior crossbite, two patients; anterior open bite, one patient.

### 2.3. Surgical Procedure

The surgical procedure was performed according to Dibart et al. [7] under local anesthesia with 4% articaine (Ubistesin forte; 3M ESPE, St. Paul, MN, USA). One hour before surgery, 1 g of amoxicillin (Ospamox; Sandoz, Holzkirchen, Austria) and 200 mg of ibuprofen (Ibuprofen Hasco; Hasco-Lek, Wrocław, Poland) were administered. After performing sulcular and vertical incisions in mucosa between the roots of the teeth, the mucoperiosteal flap was elevated as a tunnel, and then linear cuts were made through the buccal cortical plate using the OT7S-3 ultrasound tips of the piezosurgery device (Mectron, Carasco, Italy). Vertical cuts extended from the apical region of the roots to 2–3 mm from the crest. In the case of evident roots’ proximity, vertical cuts were abandoned. Then, the flap was sutured with resorbable monofilament 5.0 sutures (Biosyn; Medtronic, Mounds View, MN, USA). Mouth rinsing with chlorhexidine (Eludri; Pierre Fabre Sante, Paris, France) twice per day was prescribed as well as gentle tooth brushing in the surgical area for 2 weeks. The sutures were removed 14 days post-op (if necessary).

### 2.4. Orthodontic Treatment

The treatment was carried out with Straight-wire technique using the passive self-ligating brackets (System H4; Ortho Classic, McMinnville, OR, USA) that were bonded directly before the surgery. Following the surgery, orthodontic wire 0.014 Cooper Ni-Ti (American Orthodontics, Sheboygan, WI, USA) was mounted. The orthodontic appointments were scheduled twice monthly for the first 3 months and then once a month. The arches were fully leveled and aligned by using increasing sizes of nickel–titanium alloy archwires. The subsequent stages of treatment involved the use of 0.018 Cooper Ni-Ti wires and rectangular ones (American Orthodontics, Sheboygan, WI, USA). Subsequent stages of treatment included the use of Ni-Ti Cooper 0.016, 0. 018, and rectangular arches 0.016 × 0.25, 0.017 × 0.025, 0.018 × 0.025, and 0.019 × 0.025 (American Orthodontics, Sheboygan, WI, USA) to expand the dental arches, followed by steel arches 0.016 × 0.022, 0.017 × 0.025, 0.018 × 0.025, 0.019 × 0.025, and 0.021 × 0.025 (Ortho Classic, McMinnville, OR, USA) to achieve adequate torc and stabilizing effects of orthodontic therapy.

### 2.5. CBCT Examination

CBCT scans were taken twice, before treatment for diagnostic purposes and after treatment completion before appliance removal, in order to evaluate the position of the roots in the alveolar process. Technical parameters of the examination were set automatically according to the CBCT machine software (Pax-i3D; Vatech, Gyeonggi-do, Korea) depending on the individual characteristics of the patient. The scans were taken with the field of view size 10 × 8 cm^2^; voxel size was 0.2 mm; CBCT images’ format was DICOM; monitor size was 21.3″; and monitor resolution was 1200 × 1600. All files were exported and reconstructed using software (EzDent-i; Vatech, Gyeonggi-do, Korea) with a 0.1-mm slice interval. Each image of every tooth was oriented in a way so the whole root would be visible in one sagittal plane. In molars, the mesial and distal buccal roots were evaluated separately. In cases when the tested parameter could not be reliably evaluated, the measurement was discontinued [24].

Evaluation of the CBCT scans was performed by a well-calibrated clinician. Calibration consisted of examining the CBCT scans of five patients twice, 48 h apart.

**Figure 1:**
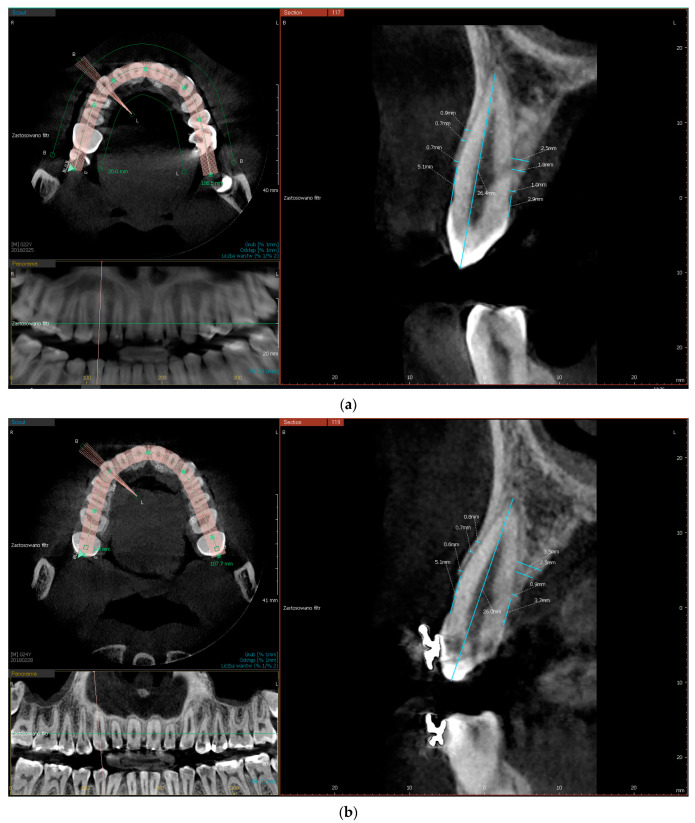
(**a**) CBCT scan of tooth No. 13 taken before treatment. CEJ-B = 5.1 mm, CEJ-P = 2.9, buccal plate thickness 0.5 mm = 0.7 mm, buccal plate thickness 3.5 mm = 0.7 mm, buccal plate thickness 5 mm = 0.9 mm 2.9 mm, palatal plate thickness 0.5 mm = 1 mm, palatal plate thickness 3.5 mm = 1.8 mm, palatal plate thickness 5 mm = 2.5 mm. (**b**) CBCT scan of tooth No. 13 taken after treatment. CEJ-B = 5.1 mm, CEJ-P = 3.7 mm, buccal plate thickness 0.5 mm = 0.6 mm, buccal plate thickness 3.5 mm = 0.7 mm, buccal plate thickness 5 mm = 0.8 mm, palatal plate thickness 0.5 mm = 0.9 mm, palatal plate thickness 3.5 mm = 2.5 mm, palatal plate thickness 5 mm = 3.5 mm.

The following radiographic parameters were measured for each root:

- buccal/palatal bone plate thickness, measured perpendicular to the long axis of the tooth at three locations, 0.5 mm, 3.5 mm, and 5 mm from the edge of alveolar process
- CEJ-C (cemento-enamel junction-crest), the distance between CEJ and the edge of buccal/palatal bone plate (CEJ-B/CEJ-P) measured parallel to the long axis of the tooth at the middle of the tooth crown. A distance of more than 2 mm was considered bone dehiscence (Figure 1a,b).

### 2.6. Model Measurements

The arch extension range was measured on the dental casts. Measurements were made between points on the palatal surfaces of the canines, premolars, and molars. The measurement point was in the central part of the palatal area in the most apical aspect.

### 2.7. Statistical Analysis

In statistical analysis, normal distribution was verified by the Kolmogorov–Smirnov test combined with the Lilliefors amendment. The Wilcoxon matched pairs test was used to compare dependent variables of the radiographic examination. The reproducibility of the CBCT scans’ examination was calculated using the Kappa test. Pearson’s linear correlation or Spearman’s rank correlation (depending on whether the assumptions about normality of distribution were met) were used to assess the relationship between the parameters. The results are given as mean ± standard deviation and 95% confidence intervals. The results were considered statistically significant at *p* < 0.05. The Statistica 12.0 package (StatSoft, Tulsa, OK, USA) was used for all calculations.

## 3. Results

The study involved 13 women aged 23 to 52 and seven men aged 22 to 56. The age of most patients was between 30 and 50 years old. Three patients each were under 30 and over 50 years of age.

The evaluation included 188 teeth (231 roots) in maxilla: 44 first and second molars (43 mesial roots, 44 distal roots), 70 first and second premolars, 35 canines, and 39 central and lateral incisors.

CBCT analysis showed that before the treatment, the buccal bone plate margin on most incisors (87.1%) was at least 2 mm from CEJ. After treatment, the percentage of incisors with bone margin above 2 mm from CEJ decreased to 66.6%. For canines and premolars, the percentage of teeth with CEJ-B ≤2 mm was 71.4% and 64.2%. After treatment, the percentage of teeth with CEJ-B >2 mm increased to 80% on canines and decreased to 67.1% on premolars. On mesial and distal molar roots, the CEJ-B also exceeded 2 mm for most teeth (67.4%, 52.2%). After treatment, the number of molars where the distance of CEJ-B exceeded 2 mm remained comparable to the baseline (65.1%, 54.5%).

At the baseline, the thickness of the buccal bone plate measured 0.5 mm from the margin and was <1 mm in most teeth (incisors, 89.7%; canines, 97.1%; premolars, 57.1%; mesial molars’ roots, 69.7%; and distal molars’ roots, 38.6%). After treatment there was an increase in the number of teeth with the buccal plate <1 mm only in premolars to 78.5%. In other regions the percentage of teeth with buccal plate <1 mm was incisors, 84.6%; canines, 91.4%; mesial roots of molars, 69.7%; and distal root of molars, 34/1%. Table 1 and Table 2 contain the distribution of teeth depending on the buccal plate margin distance from CEJ and the thickness of the buccal bone plate measured 0.5 mm from CEJ.

After treatment there were slight changes in CEJ-C and thickness of the buccal and palatal plates for all dental groups except for incisors and premolars, where certain changes were significant. CEJ-B increased by 1.43 mm on premolars (*p* = 0.023) and CEJ-P by 1.65 mm on incisors (*p* < 0.0001) and by 0.31 mm on premolars (*p* = 0.037).

On the incisors’ buccal bone plate, the width increased significantly by 0.2 mm (*p* = 0.002) and 0.44 mm (*p* < 0.0001) at 3.5-mm and 5-mm measurement points. Palatal plate width decreased by 0.23 mm (*p* = 0.027) and 0.28 mm (*p* = 0.018) in the same measurement points. On premolars, the buccal plate width decreased in the three measuring points by 0.27 mm, 0.37 mm, and 0.25 mm (*p* < 0.0001), respectively, but the palatal plate width increased by 0.2 mm (*p* = 0.001) in the measurement 0.5 mm from the crest (Table 3, Table 4, Table 5, Table 6, Table 7 and Table 8). Figure 1a,b shows an example of CBCT scans of one of the patients taken before and after treatment.

The mean size of arch expansion measured on the dental casts was 1.45 ± 2.16 mm on canines, 1.99 ± 1.69 mm on premolars, and 0.15± 0.44 mm on molars. There was no correlation between the arch expansion and CEJ-B and buccal bone thickness values (Table 9).

Intraexaminer reliability of CBCT examination was high, which indicates the repeatability of the measurements. Table 10 shows the Kappa test for each parameter calculated from a comparison of the two examinations of five patients performed 48 h apart.

## 4. Discussion

The purpose of this study was to find the answer to the question if there are changes in the width and height of the buccal and palatal bone plates after piezocision-assisted orthodontic expansion of the upper arch. It has not been shown that there is a significant deterioration of radiographic parameters in the incisor, canine, and molar regions after this procedure. However, it has been shown that significant deterioration of radiographic parameters occurs in the premolar area. These data are all the more meaningful because they were found in patients in whom the baseline values of primary parameters, i.e., thickness and height of buccal bone plate, were typical of the thin phenotype. Initial examination revealed that, at the measuring point located 0.5 mm from the bone crest, the buccal plate thinner than 1 mm was found on 89.7% of incisors, 97.1% of canines, 57.1% of premolars, 69.7% of mesial molars’ roots, and 38.6% of distal molars’ roots. Furthermore, this examination concluded that bone dehiscences were most common on premolars and incisors.

The baseline results of the present study are in agreement with the data in the literature, which showed that the majority of teeth exhibited less than 1 mm buccal bone plate, e.g., 62.9% at 4 mm from CEJ and 80.1% in the middle of root, or no bone wall, respectively, in 25.7% and 10.0%. A thick, ≥1 mm, bone plate was detected in 11.4% of teeth in the first and in 9.8% of teeth in the second aforementioned measurement points. Additionally, a statistically significant decrease in buccal bone plate thickness from the first premolars to the central incisors was noted [24]. Measurements performed by other authors in three measuring points, which were 1, 3, and 5 mm from the bone margin, showed that ranges of buccal plate thickness on premolars were 0.36–3.62 mm, 0.23–3.77 mm, and 0.19–2.92 mm. For molars, ranges of bone thickness in these three measurement points were, respectively, 0.4–3.47 mm, 0.14–4.97 mm, and 0.07–6.19 mm. Analyzing the thickness of the buccal plate separately on mesial and distal roots of molars, some authors found that buccal plate thickness on the mesial roots of molars was similar to that on premolars [25]. They also revealed that buccal plate dehiscences most often occurred on mesial roots of molars and on roots of premolars [26]. However, they did not find any correlation between buccal bone dehiscence occurrence, age, and gender [25,26].

The presence of buccal plate dehiscences in patients with Classes I and II division 1 malocclusion was studied by Evangelista et al. [22]. For bone plate dehiscence, they accepted the situation where plate margin was more than 2 mm apically from CEJ. They revealed that buccal bone dehiscences most often occur on canines, first premolars, and molars (18.73%, 18.45%, 18.27%), next on lateral incisors and second molars (13.35%, 11.26%), and the least on second premolars and central incisors (10.48%, 9.46%). Additionally, they found that bone dehiscences more often occur in patients with Angle’s Class I than with Class II division 1, and that they are not related to the facial type.

Analysis of the parameters assessed in the present study showed that only minor changes in their value occurred after the piezocision-assisted orthodontic treatment. A significant reduction in buccal plate width and bone dehiscence enlargement was observed only on premolars. The explanation for this observation may be that in this region the expansion of the arch was most prominent. The extent of the arch expansion is also underlined by some authors as a likely factor of bone loss in the premolar region [27]. However, in our study, no correlation was found between the size of the arch expansion and the thickness of the buccal plate and the size of bone dehiscence.

There are very little data in available literature about CBCT assessment after piezocision-assisted orthodontic treatment. There are only case reports and only one article presenting a randomized, controlled study, which compared classic orthodontic treatment with piezocision-assisted orthodontic treatment. Authors of this study concluded that, after both types of treatment, the number of bone dehiscences and fenestrations did not increase significantly. Moreover, the thickness of the buccal plate and the buccolingual dimension of alveolar crest did not change significantly in both groups. However, the authors did not present any numeric data [28].

Quite different data came out of a study concerning classic orthodontic expansion of the upper arch with use of self-ligation brackets (Damon 3MX) in treatment of crowding. Indeed, it was shown that this procedure may be involved in the worsening of dentition periodontal status. CBCT evaluation revealed that non-extraction alignment led to horizontal and vertical bone loss at the incisors and mesial root of the first molars. Buccal plate thickness measured 3 mm from CEJ decreased significantly, by 0.2 mm on incisors and by 0.6 mm on the mesial root of first molars. Bone plate width on these roots decreased by 0.5 mm also in measurements taken 6 mm from CEJ. Distances of CEJ-B did not differ significantly before and after treatment on certain teeth groups; but, still, the surface area of the buccal plate decreased significantly on incisors by 1.2 mm^2^ and by mesial roots of the first molars by 4.3 mm^2^. Moreover, some authors noted that an initially thinner buccal plate was correlated with greater apical loss of bone in the incisors’ region [27].

According to other authors, after using the same brackets, the surface area of the buccal plate measured on the first right and left premolars decreased by 3.4 mm^2^ and 2.7 mm^2^. Additionally, the distance of CEJ-B noticeably increased by the majority of the teeth (12/21). In 10 of 21 patients, loss of bone plate height did not exceed 0.7 mm; but in two it did. The use of other-active, self-ligating brackets (In-Ovation R) similarly influenced bone remodeling. The surface area of the buccal plate decreased on the left and right first premolars by 2.3 mm^2^. Changes in marginal bone level in relation to CEJ took place in 11 out of 20 patients, but loss of bone height below 0.7 mm occurred in six patients and above 0.7 mm in five patients [29].

The present study showed that the thickness of the palatal plate in three measuring points as well as dehiscence CEJ-P were comparable before and after treatment in all groups of teeth. The only significant changes were an increase of palatal plate thickness on premolars measured 0.5 mm from CEJ, a decrease of this parameter on incisors at 3.5 mm and 5 mm measurement points, as well as an increase of palatal bony dehiscence on incisors and premolars. Similarly, no significant changes were observed by other authors who evaluated CBCT scans after slow maxillary expansion with quadhelix expanders and rapid maxillary expansion with Haas/Hyrax-type expanders. In the molar region, palatal bone thicknesses in both evaluated groups were 1.63 ± 0.55 and 1.48 ± 0.69 mm before treatment. After treatment completion, the value of this parameter increased insignificantly to 2.07 ± 1.44 (difference—0.44) and 1.78 ± 0.87 (difference—0.29) [30].

The literature data presented above are characterized by a significant discrepancy in the results depending on the procedures performed. However, regardless of the type of procedure, the values of change in radiographic parameters are rather small. Therefore, one should remember that these differences (in plus and in minus) may not necessarily come out of the treatment but of measurement inaccuracy. When performing CBCT analysis, care should be taken on factors that can possibly affect its reliability. The crucial factor hindering the analysis is anatomical variety of certain structures. It seems that more accurate evaluation of CT scans is possible when the alveolar bone is 0.5 mm thick. Additionally, a visible periodontal ligament space up to 0.2 mm was considered to improve the reliability of the measurement of buccal or lingual bone plates [19,20]. Such problems depend on CBCT machine parameters (numbers of shades, signal-to-noise ratio, voxel size) [31,32]. Reduction of voxel size to 0.125 mm improves significantly examination reading, but demands higher radiation dose, which is not recommended for orthodontic indications [32]. Repeatability of CBCT examination is also an important aspect. Scans’ analysis in our study was performed by a calibrated examiner, as evidenced by high Kappa coefficient value, which indicates the repeatability of the study.

After analyzing the data presented above regarding piezocision-assisted orthodontic arch expansion, it must be emphasized that this topic is still a niche one. As far as we know, there are no publications in the currently available literature that evaluate bone after this type of treatment. Therefore, our study brings new radiographic data regarding changes in the cortical bone. It has been shown that both favorable and unfavorable changes in the thickness and height of the buccal and palatal bone plates take place after treatment. These changes mostly do not reach statistical significance. The only teeth on which thickness and height of the buccal plate significantly decreased were premolars. Therefore, this region should be analyzed in detail before treatment in terms of anatomical conditions and the extent of arch expansion.

This observation is particularly important in the aspect of the indications to more invasive surgery like corticotomy with additional alveolar ridge augmentation. When deciding on corticotomy with augmentation, one should also keep in mind some limitations of these procedures. Indeed, after the augmentation a wider buccal plate is generally visible on CBCT, but this image is not synonymous with histological results. Currently, there are no recommendations as to the type of biomaterials and barrier membranes used in such procedures [10,33,34,35,36,37,38,39]. It does not change the fact that, irrespective of the material used, during augmentation procedure after corticotomy/piezocision it is difficult to provide the conditions needed for regeneration [40]. Therefore, taking into consideration the lack of histologically confirmed bone regeneration and the unpredictability of the scope and place of bone gain on the one hand and the high burden of surgery itself on the other hand, consideration should be given to the choice of surgery prior to orthodontic treatment. Therefore, taking into account minimally invasive techniques like piezocision before orthodontic treatment seems reasonable.

The present study was designed as a case series, which can be seen as a limitation of this study. Assumption of this research model was to identify possible negative changes in bony plates that may be considered as risk factors for gingival recession. However, this model did not provide a chance to answer the question whether the piezocision resulted in additional changes in the radiographic parameters compared to conventional orthodontic treatment. However, the abandonment of the control group was consciously undertaken and was dictated by ethical considerations. Establishing a control group could have doomed patients to potential complications after arch expansion–gingival recessions. Most patients had a thin phenotype, which, according to current knowledge, requires gingival augmentation before treatment [1,2,5]. Performing such a procedure could affect the results of the planned radiographic examination. We believe that the ethical aspect was an overriding factor in the choice of study model. This does not change the fact that randomized studies comparing classic orthodontic arch expansion with piezocision-assisted expansion are the most scientifically desirable.

## 5. Conclusions

Given the limitations of the study, it can be concluded that piezocision-assisted orthodontic maxillary arch expansion does not cause evident negative changes of cortical bone plates except for the premolar region. Therefore, premolars may be at greater risk of buccal plate loss than other teeth.

## Figures and Tables

**Table 1:** Root distribution depending on the distance between the buccal bone plate margin and CEJ (CEJ-B) before and after orthodontic treatment (Mm, mesial root of molar; Md, distal root of molar).

CEJ-B	Incisors		Canines		Premolars		Mm		Md	
	Baseline	Post-op	Baseline	Post-op	Baseline	Post-op	Baseline	Post-op	Baseline	Post-op
≤2 mm	5	13	10	7	25	23	14	15	21	20
>2 mm	34	26	25	28	45	47	29	28	23	24

**Table 2:** Root distribution depending on the thickness of the buccal bone plate at the most coronal measurement point (0.5 mm from the crest) before and after orthodontic treatment (Mm, mesial root of molar; Md, distal root of molar).

Buccal Plate Thickness	Incisors		Canines		Premolars		Mm		Md	
	Baseline	Post-op	Baseline	Post-op	Baseline	Post-op	Baseline	Post-op	Baseline	Post-op
<1 mm	35	33	34	32	40	55	30	30	17	15
≥1 mm	4	6	1	3	30	15	13	13	27	29

**Table 3:** Distribution of CBCT parameters for incisors before and after orthodontic treatment.

Parameter	Timing	Mean ± SD	Difference	95% Cl	*p*
Buccal plate thickness 0.5 mm	Baseline	0.68 ± 0.21	0.03	0.07 (0.62; 0.75)	0.299
	Post-op	0.71 ± 0.24		0.07 (0.64; 0.79)	
Buccal plate thickness 3.5 mm	Baseline	0.65 ± 0.40	0.2	0.13 (0.52; 0.77)	0.002
	Post-op	0.85 ± 0.51		0.16 (0.69; 1.01)	
Buccal plate thickness 5 mm	Baseline	0.52 ± 0.37	0.44	0.12 (0.40; 0.63)	<0.0001
	Post-op	0.96 ± 0.80		0.25 (0.71; 1.22)	
Palatal plate thickness 0.5 mm	Baseline	0.74 ± 0.29	0.02	0.09 (0.65; 0.83)	0.587
	Post-op	0.72 ± 0.33		0.10 (0.62; 0.82)	
Palatal plate thickness 3.5 mm	Baseline	1.99 ± 0.84	0.23	0.26 (1.73; 2.26)	0.027
	Post-op	1.76 ± 0.78		0.24 (1.52; 2.01)	
Palatal plate thickness 5 mm	Baseline	2.72 ± 1.07	0.28	0.34 (2.39; 3.06)	0.018
	Post-op	2.44 ± 1.04		0.33 (2.11; 2.77)	
CEJ-C buccal	Baseline	2.67 ± 1.18	0.09	0.37 (2.30; 3.05)	0.87
	Post-op	2.76 ± 1.19		0.37 (2.39; 3.13)	
CEJ-C palatal	Baseline	1.67 ± 0.78	1.65	0.24 (1.43; 1.91)	<0.0001
	Post-op	3.32 ± 2.58		0.81 (2.51; 4.13)	

CEJ-C, cemento-enamel junction-crest.

**Table 4:** Distribution of CBCT parameters for canines before and after orthodontic treatment.

Parameter	Timing	Mean ± SD	Difference	95% Cl	*p*
Buccal plate thickness 0.5 mm	Baseline	0.67 ± 0.28	0.05	0.08 (0.20; 0.36)	0.257
	Post-op	0.62 ± 0.27		0.08 (0.20; 0.35)	
Buccal plate thickness 3.5 mm	Baseline	0.61 ± 0.42	0.02	0.12 (0.30; 0.53)	0.706
	Post-op	0.63 ± 0.52		0.14 (0.37; 0.66)	
Buccal plate thickness 5 mm	Baseline	0.58 ± 0.61	0.02	0.17 (0.44; 0.78)	0.226
	Post-op	0.60 ± 0.51		0.14 (0.37; 0.65)	
Palatal plate thickness 0.5 mm	Baseline	0.89 ± 0.59	0.02	0.17 (0.43; 0.76)	0.235
	Post-op	0.91 ± 0.38		0.11 (0.27; 0.48)	
Palatal plate thickness 3.5 mm	Baseline	1.95 ± 1.14	1.48	0.32 (0.82; 1.46)	0.801
	Post-op	3.43 ± 0.83		0.23 (0.60; 1.07)	
Palatal plate thickness 5 mm	Baseline	2.66 ± 1.58	0.77	0.44 (1.14; 2.03)	0.722
	Post-op	3.43 ± 1.17		0.33 (0.84; 1.50)	
CEJ-C buccal	Baseline	3.43 ± 2.19	0.49	0.61 (2.82; 4.04)	0.644
	Post-op	3.92 ± 3.12		0.87 (2.24; 3.99)	
CEJ-C palatal	Baseline	2.80 ± 1.59	0.64	0.45 (1.15; 2.04)	0.152
	Post-op	3.44 ± 2.20		0.62 (1.58; 2.81)	

CEJ-C, cemento-enamel junction-crest.

**Table 5:** Distribution of CBCT parameters for premolars before and after orthodontic treatment.

Parameter	Timing	Mean ± SD	Difference	95% Cl	*p*
Buccal plate thickness 0.5 mm	Baseline	0.97 ± 0.40	0.27	0.09 (0.30; 0.49)	<0.0001
	Post-op	0.70 ± 0.39		0.09 (0.30; 0.48)	
Buccal plate thickness 3.5 mm	Baseline	0.93 ± 0.65	0.37	0.15 (0.49; 0.80)	<0.0001
	Post-op	0.56 ± 0.40		0.09 (0.31; 0506)	
Buccal plate thickness 5 mm	Baseline	0.84 ± 0.74	0.25	0.17 (0.57; 0.92)	<0.0001
	Post-op	0.59 ± 0.52		0.12 (0.40; 0.64)	
Palatal plate thickness 0.5 mm	Baseline	0.87 ± 0.30	0.2	0.07 (0.23; 0.37)	0.001
	Post-op	1.07 ± 0.39		0.09 (0.30; 0.48)	
Palatal plate thickness 3.5 mm	Baseline	1.79 ± 0.71	0.14	0.17 (0.54; 0.87)	0.097
	Post-op	1.93 ± 0.76		0.18 (0.58; 0.94)	
Palatal plate thickness 5 mm	Baseline	2.49 ± 1.02	0.02	0.24 (0.78; 1.26)	0.873
	Post-op	2.47 ± 1.04		0.24 (0.80; 1.29)	
CEJ-C buccal	Baseline	2.73 ± 1.26	1.43	0.29 (2.43; 3.02)	0.023
	Post-op	4.16 ± 4.01		0.94 (3.07; 4.95)	
CEJ-C palatal	Baseline	2.32 ± 1.04	0.31	0.24 (0.80; 1.29)	0.037
	Post-op	2.63 ± 1.04		0.24 (0.79; 1.28)	

CEJ-C, cemento-enamel junction-crest.

**Table 6:** Distribution of CBCT parameters for molars before and after orthodontic treatment.

Parameter	Timing	Mean ± SD	Difference	95% Cl	*p*
Buccal plate thickness 0.5 mm	Baseline	0.99 ± 0.48	0.12	0.10 (0.38; 0.58)	0.177
	Post-op	1.11 ± 0.58		0.12 (0.46; 0.69)	
Buccal plate thickness 3.5 mm	Baseline	1.13 ± 0.89	0.09	0.18 (0.71; 1.07)	0.375
	Post-op	1.03 ± 0.77		0.16 (0.61; 0.92)	
Buccal plate thickness 5 mm	Baseline	1.16 ± 1.11	0.12	0.22 (0.88; 1.33)	0.439
	Post-op	1.04 ± 0.87		0.18 (0.69; 1.04)	
Palatal plate thickness 0.5 mm	Baseline	1.59 ± 0.98	0.07	0.20 (0.78; 1.18)	0.159
	Post-op	1.66 ± 0.96		0.19 (0.77; 1.16)	
Palatal plate thickness 3.5 mm	Baseline	2.91 ± 2.0	0.14	0412 (1.60; 2.41)	0.777
	Post-op	2.77 ± 2.12		0.43 (1.69; 2.55)	
Palatal plate thickness 5 mm	Baseline	3.36 ± 2.52	0.19	0.51 (2.01; 3.03)	0.893
	Post-op	3.17 ± 2.61		0.53 (2.08; 3.14)	
CEJ-C buccal	Baseline	2.98 ± 2.07	0.33	0.42 (2.56; 3.39)	0.870
	Post-op	2.65 ± 1.58		0.32 (1.26; 1.90)	
CEJ-C palatal	Baseline	2.33 ± 1.03	0.35	0.21 (0.82; 1.23)	0.077
	Post-op	2.68 ± 1.74		0.35 (1.39; 2.09)	

CEJ-C, cemento-enamel junction-crest.

**Table 7:** Distribution of CBCT parameters for mesial molars’ roots before and after orthodontic treatment.

Parameter	Timing	Mean ± SD	Difference	95% Cl	*p*
Buccal plate thickness 0.5 mm	Baseline	0.85 ± 0.34	0.15	0.010(0.75; 0.95)	0.653
	Post-op	0.90 ± 0.43		0.13 (0.77; 1.03)	
Buccal plate thickness 3.5 mm	Baseline	0.96 ± 0.87	0.17	0.126(0.70; 1.21)	0.106
	Post-op	0.79 ± 0.75		0.22 (0.57; 1.01)	
Buccal plate thickness 5 mm	Baseline	1.04 ± 1.09	0.26	0.32 (0.72; 1.37)	0.036
	Post-op	0.78 ± 0.75		0.22 (0.56; 1.01)	
Palatal plate thickness 0.5 mm	Baseline	2.08 ± 1.03	0.07	0.31 (1.77; 2.38)	0.687
	Post-op	2.15 ± 1.05		0.31 (1.84; 2.46)	
Palatal plate thickness 3.5 mm	Baseline	4.41 ± 1.49	0.27	0.44 (3.97; 4.85)	0.767
	Post-op	4.14 ± 1.88		0.56 (3.58; 4.69)	
Palatal plate thickness 5 mm	Baseline	5.27 ± 1.89	0.41	0.46 (4.71; 5.83)	0.980
	Post-op	4.86 ± 2.30		0.68 (4.18; 5.54)	
CEJ-C buccal	Baseline	3.02 ± 1.94	0.12	0.57 (2.45; 3.59)	0.468
	Post-op	2.90 ± 1.89		0.56 (2.34; 3.46)	
CEJ-C palatal	Baseline	2.16 ± 0.79	0.43	0.23 (1.92; 2.39)	0.072
	Post-op	2.59 ± 1.75		0.52 (2.08; 3.11)	

CEJ-C, cemento-enamel junction-crest.

**Table 8:** Distribution of CBCT parameters for distal molars’ roots before and after orthodontic treatment.

Parameter	Timing	Mean ± SD	Difference	95% Cl	*p*
Buccal plate thickness 0.5 mm	Baseline	1.13 ± 0.56	0.19	0.16 (0.97; 1.30)	0.164
	Post-op	1.32 ± 0.62		0.18 (1.14; 1.50)	
Buccal plate thickness 3.5 mm	Baseline	1.33 ± 0.92	0.02	0.27 (1.06; 1.60)	0.895
	Post-op	1.31 ± 0.74		0.22 (1.09; 1.53)	
Buccal plate thickness 5 mm	Baseline	1.32 ± 1.15	0	0.34 (0.98; 1.66)	0.581
	Post-op	1.32 ± 0.93		0.28 (1.05; 1.60)	
Palatal plate thickness 0.5 mm	Baseline	1.07 ± 0.61	0.11	0.18 (0.89; 1.25)	0.038
	Post-op	1.18 ± 0.51		0.15 (1.02; 1.33)	
Palatal plate thickness 3.5 mm	Baseline	1.38 ± 1.08	0.02	0.32 (1.07; 1.70)	0.890
	Post-op	1.40 ± 1.28		0.38 (1.02; 1.78)	
Palatal plate thickness 5 mm	Baseline	1.41 ± 1.25	0.02	0.37 (1.04; 1.78)	0.845
	Post-op	1.43 ± 1.51		0.45 (0.99; 1.88)	
CEJ-C buccal	Baseline	2.91 ± 2.19	0.51	0.65 (2.26; 3.55)	0.277
	Post-op	2.40 ± 1.13		0.33 (2.07; 2.73)	
CEJ-C palatal	Baseline	2.51 ± 1.19	0.26	0.35 (2.16; 2.87)	0.454
	Post-op	2.77 ± 1.72		0.51 (2.26; 3.27)	

CEJ-C, cemento-enamel junction-crest.

**Table 9:** The correlation between the arch expansion and CEJ-B and buccal bone thickness values.

Correlation	R	*p*
Canines		
arch expansion—CEJ-B	0.07	0.702
arch expansion—buccal plate thickness	0.08	0.658
Premolars		
arch expansion—CEJ-B	0.07	0.583
arch expansion—buccal plate thickness	−0.13	0.281
Molars		
arch expansion—CEJ-B	0.27	0.071
arch expansion—buccal plate thickness	−0.20	0.186

**Table 10:** Intraexaminer reliability of radiographic parameters.

Parameter	Kappa Coefficient	*p*
Buccal plate thickness 0.5 mm	0.97	<0.0001
Buccal plate thickness 3.5 mm	0.99	<0.0001
Buccal plate thickness 5 mm	0.99	<0.0001
Palatal plate thickness 0.5 mm	0.98	<0.0001
Palatal plate thickness 3.5 mm	0.99	<0.0001
Palatal plate thickness 5 mm	0.99	<0.0001
CEJ-C buccal	0.96	<0.0001
CEJ-C palatal	0.94	<0.0001

## Data Availability

The data presented in this study are available on request from the corresponding author. The data are not publicly available due to privacy restrictions.
